# Association Between Gestational Blood Pressure Trajectories and Postpartum Normotension Recovery in Hypertensive Disorders: Retrospective Cohort Study

**DOI:** 10.2196/89295

**Published:** 2026-04-30

**Authors:** Zhijiang Liang, Huili Wei, Xiaojun Xu, Rong Xu, Xinyue Yang, Jinlian Dong, Xiaoyan Fan, Yukun Chen, Meiling Feng, Xin Zhou, Yijun Cai, Lijuan Lv

**Affiliations:** 1 Department of Medical Information and Statistics Guangdong Women and Children Hospital Guangzhou, Guangdong China; 2 Department of Public Health School of Medicine Jinan University Guangzhou, Guangdong China; 3 Department of Cardiology Tianjin Medical University General Hospital Tianjin China; 4 Medical Genetic Center and Department of Obstetrics Guangdong Women and Children Hospital Guangzhou, Guangdong China; 5 Medical Genetic Center and Department of Obstetrics Gaozhou Hospital, Guangdong Women and Children Hospital Gaozhou, Guangdong China

**Keywords:** blood pressure trajectory, group-based trajectory modeling, hypertensive disorders of pregnancy, postpartum BP recovery status

## Abstract

**Background:**

Hypertensive disorders of pregnancy (HDP) may cause lasting vascular, cardiac, and renal damage, potentially increasing the risk of postpartum cardiovascular disease.

**Objective:**

This study aimed to examine the association between gestational blood pressure (BP) trajectories in HDP and the risk of unrecovered BP at 6 weeks post partum.

**Methods:**

A total of 3162 women with HDP were obtained from the antenatal care and the postpartum follow-up information system, between January 1, 2018, and December 31, 2024. Of the 3162 women included, 1674 had gestational hypertension, 607 had preeclampsia, 246 had chronic hypertension with superimposed preeclampsia, and 635 had chronic hypertension. Group-based trajectory modeling was used to fit systolic BP (SBP), diastolic BP (DBP), and mean arterial pressure (MAP) trajectories during pregnancy. Modified Poisson regression was used to assess the association between gestational BP trajectories and the risk of unrecovered BP at 6 weeks post partum.

**Results:**

Trajectories of SBP, DBP, and MAP during pregnancy were significantly associated with unrecovered BP at 6 weeks post partum. For gestational hypertension, those with the high-consistent rise (adjusted relative risk [aRR] 2.493, 95% CI 1.093-5.689) and high-late surge SBP trajectories (aRR 4.535, 95% CI 1.884-10.917) were associated with a significantly increased risk of BP nonrecovery at 6 weeks post partum. Similar associations were observed for DBP and MAP. For chronic hypertension with superimposed preeclampsia, women with high-late surge in SBP (aRR 2.792, 95% CI 1.081-7.214), DBP (aRR 4.043, 95% CI 1.327-12.324), or MAP (aRR 4.018, 95% CI 1.462-11.045) had a significantly increased risk of BP nonrecovery at 6 weeks post partum. Among women with chronic hypertension, those with the high-consistent rise trajectories of SBP (aRR 2.557, 95% CI 1.256-5.207), DBP (aRR 3.862, 95% CI 1.673-8.913), and MAP (aRR 3.714, 95% CI 1.682-8.201) had a significantly increased risk of BP nonrecovery at 6 weeks post partum. Among women with preeclampsia, only high-consistent rise SBP trajectory remained significantly associated with unrecovered BP post partum (aRR 3.355, 95% CI 1.140-9.873). The high-consistent rise and high-late surge trajectories of SBP, DBP, and MAP in gestational hypertension started at similar initial levels and crossed at approximately 22 weeks of gestation.

**Conclusions:**

The gestational BP trajectories in women with HDP are positively associated with the risk of unrecovered BP at 6 weeks post partum. Early identification of women at high risk for poor postpartum BP recovery through BP trajectory analysis may have important clinical implications for improving long-term cardiovascular outcomes in this population.

## Introduction

Hypertensive disorders of pregnancy (HDP) encompass a spectrum of conditions characterized by elevated blood pressure (BP) during gestation, affecting approximately 5%-12% of pregnancies. Based on the type of disease, HDP include gestational hypertension (GH), preeclampsia-eclampsia, chronic hypertension (CHTN) with superimposed preeclampsia, and CHTN [1,2]. HDP pose a serious threat to maternal health and are among the leading causes of maternal morbidity and mortality worldwide [3]. Women with a history of HDP are at increased risk of developing cardiovascular disease (CVD), the leading cause of death among women [4,5].

Although BP in most patients with HDP gradually normalizes after delivery, the exact time to recovery remains inconsistent across studies [6-8]. The vascular, cardiac, and renal damage caused by HDP may persist beyond pregnancy [9], potentially resulting in persistent postpartum hypertension [10] and an increased long-term risk of adverse cardiovascular events [9]. Postpartum hypertension after preeclampsia may take up to 2 years to resolve [11]. A study found that 39% of women still had hypertension three months after delivery, while this figure dropped to 18% after 2 years [11]. Therefore, when studying remaining hypertension after pregnancies complicated by hypertensive disorders, it is important to take this time frame into consideration [12].

Evidence suggests that the first 6 weeks post partum represent a critical window for cardiovascular risk intervention [8,13]. This aligns with Chinese clinical guidelines, which recommend a postpartum follow-up visit at 6 weeks for women with HDP [14,15]. The Hypertension Canada guidelines recommend continued hypertension management and monitoring until at least 6 weeks post partum in women with cardiovascular risk factors [16]. From 2014 to 2017, HDP, including preeclampsia, accounted for 6.8% of pregnancy-related deaths in the United States, with 65% occurring within 6 weeks post partum [17]. Thus, assessment of BP recovery at this time point is of significant clinical relevance.

While clinical risk factors for impaired postpartum BP recovery are generally considered to include HDP subtype, BP at onset, and maximum antepartum systolic BP (SBP) [18-20], these single-time measurements are susceptible to variability. In contrast, BP trajectories derived from repeated measurements better capture cardiovascular adaptation during pregnancy and are stronger predictors of adverse outcome. For instance, first-trimester BP trajectories correlate strongly with later hypertension [21]. Thus, analyzing BP trajectories rather than isolated readings may offer superior insight into postpartum BP recovery in HDP.

In this study, we classified patients with HDP into 4 subtypes: GH, preeclampsia, CHTN with superimposed preeclampsia, and CHTN [14]. We modeled trajectories of SBP, diastolic BP (DBP), and mean arterial pressure (MAP) across multiple time points during pregnancy within each subtype to evaluate the association between BP trajectories during pregnancy and the risk of unrecovered BP at 6 weeks post partum. By elucidating the heterogeneity of BP changes across HDP subtypes, we aim to improve risk stratification and management of HDP. Furthermore, identifying high-risk trajectory may enable early detection of women at increased risk of poor BP recovery, thereby providing a scientific basis for individualized postpartum follow-up and early intervention of future CVD.

## Methods

### Study Participants

This study was mainly conducted in the western region of Guangdong Province, China, including Zhanjiang, Maoming, and Yangjiang. Data were obtained from the antenatal care and the postpartum follow-up information system. Routine clinical data from local maternity care, generated through standard practice, were used in this study without active recruitment. Diagnostic criteria for HDP were based on the Chinese Society of Obstetrics and Gynecology guidelines [14], consistent with international standards [2,22]. GH was defined as new-onset hypertension (SBP ≥140 mm Hg and/or DBP ≥90 mm Hg) after 20 weeks of gestation without proteinuria or other features of preeclampsia. Preeclampsia was diagnosed as new-onset hypertension after 20 weeks with either significant proteinuria or, in its absence, new-onset maternal organ dysfunction or placental-fetal compromise (eg, fetal growth restriction). CHTN referred to hypertension present before 20 weeks of gestation. CHTN with superimposed preeclampsia was diagnosed in women with CHTN who developed new features of preeclampsia after 20 weeks. Baseline information was collected for pregnant women who initiated antenatal records between January 1, 2018, and December 31, 2024. Inclusion criteria were (1) diagnosis of 1 of the 4 HDP subtypes (GH, preeclampsia, CHTN with superimposed preeclampsia, or CHTN), (2) at least 1 BP measurement in each trimester, with a total of ≥3 measurements during pregnancy, and (3) available BP data at 6-week postpartum follow-up. Exclusion criteria were (1) age <18 years, (2) history of smoking or alcohol consumption, (3) multifetal pregnancy, (4) women with severe CVDs (such as valvular heart disease, myocarditis sequelae and arrhythmias requiring medication), hematologic disorders (including anemia, thrombophilia, or coagulation dysfunction), endocrine diseases (such as medication-requiring diabetes mellitus, thyroid diseases, and prolactinoma), or immune system diseases requiring hormone therapy, and (5) women with missing or incomplete BP data from antenatal or follow-up visits (only SBP or DBP available). After applying the inclusion and exclusion criteria, 3162 women were included in the final analysis. Specifically, 1674, 607, 246, and 635 pregnant women were classified as having GH, preeclampsia, CHTN with superimposed preeclampsia, and CHTN, respectively. A flowchart for the selection process of the study is provided in Figure S1 in Multimedia Appendix 1.

### BP Measurement During Pregnancy

According to the Chinese Guideline of Preconception and Prenatal Care [23], pregnant women are recommended to have regular checkups, including BP measurement, at 7 specific gestational windows. BP is measured at every antenatal visit. However, as this is a retrospective study relying on routine health information systems, not all measured BP values were consistently recorded or available in the databases. Therefore, to ensure data completeness and consistency across all included women, we applied the criterion of at least 1 recorded BP measurement per trimester (first, second, and third). The description of BP measurement frequency during pregnancy is provided in Tables S10 and S11 in Multimedia Appendix 1. SBP and DBP measurements during pregnancy were obtained from the electronic medical record system. MAP was calculated as (SBP + 2 × DBP)/3. According to the Guidelines for the Management of Hypertensive Disorders of Pregnancy (2015), participants were required to rest for at least 5 minutes prior to each BP measurement. HDP are defined as SBP ≥140 mm Hg or DBP ≥90 mm Hg on at least 2 measurements in the same arm. In women with newly detected elevated BP, a repeat measurement after ≥4 hours is required, and hypertension is diagnosed if the initial and repeat measurements both show SBP ≥140 mm Hg or DBP ≥90 mm Hg. In cases of severe hypertension, defined as SBP ≥160 mm Hg or DBP ≥110 mm Hg, the diagnosis can be confirmed by a repeat measurement after several minutes.

### Assessment of Covariates

Maternal baseline information during the study period was extracted from the electronic medical record system. Data included demographic characteristics (maternal age and education level), obstetric history (parity status, vaginal deliveries, cesarean deliveries, gravidity, abortion history, and gynecological surgery history), delivery-related factors (mode of delivery, delivery gestational age, and intrapartum BMI), and neonatal characteristics (sex, birth weight, and Apgar scores at 1, 5, and 10 minutes). Intrapartum BMI was calculated as weight at delivery (kg)/height (m^2^). Missing covariates were imputed using a random forest–based method to ensure the reliability of the analyses. Covariates adjusted in modified Poisson regression models were determined based on previous literature [21,24] and results from univariate analyses, including maternal age, education level, gravidity, delivery gestational age, parity status, mode of delivery, neonatal birth weight, and sex. As data on medication use during pregnancy and the postpartum period were not available, this variable could not be adjusted for in this analysis.

### Exposure and Outcome

In this study, the exposure was defined as BP trajectory during pregnancy. Group-based trajectory modeling (GBTM) was conducted using the TRAJ procedure (the Stata command for group‑based trajectory modeling); in Stata (version 18.0; Stata Corp) to identify SBP, DBP, and MAP trajectories. GBTM classifies individuals with similar developmental patterns within a heterogeneous population, allowing for better subgroup characterization [25,26]. As BP measurements are continuous and normally distributed, trajectory fitting used a censored normal model [27]. Since no universally accepted criteria exist for optimal model selection [28], objective indices, trajectory plot simplicity, clinical interpretability, and practical use were considered to select the final model in this study [29]. Starting from the third-order polynomial, models with various polynomial orders were repeatedly constructed and compared. Model fitting parameters for GBTM are presented in Tables S1-S8 in Multimedia Appendix 1. The objective criteria for selecting the optimal GBTM model included (1) lower absolute values of Bayesian information criterion and Akaike information criterion indicating better model fit, (2) average posterior probability of assignment >0.7 for each trajectory group, (3) odds of correct classification >5.0 for each trajectory group, (4) each trajectory group accounting for at least 5% of the sample, (5) good concordance between the posterior probability of group membership (Pj) and the actual proportion of group membership (πj), (6) minimum proportion of each trajectory group [30], and (7) statistical significance of the highest-order polynomial term in each trajectory.

The outcome was the BP recovery status at 6 weeks post partum, defined as a binary variable. BP recovery to normotension at 6 weeks post partum was defined as SBP <140 mm Hg and DBP <90 mm Hg, while BP nonrecovery to normotension was defined as SBP ≥140 mm Hg or DBP ≥90 mm Hg (1 mm Hg=0.133 kPa). BP measurements at 6 weeks post partum were measured in patients with resting HDP using an electronic sphygmomanometer. BP measured at 6 weeks post partum was obtained by a qualified physician. If the reading was abnormal (≥140/90 mm Hg), the measurement was repeated.

### Statistical Analysis

Statistical analyses were conducted using R software (version 4.4.2; R Foundation for Statistical Computing) and Stata 18.0. Statistical tests were 2-sided, and *P*≤.05 was considered statistically significant. The normality of continuous variables was assessed by the Shapiro-Wilk test, and homogeneity of variance by the Bartlett test. Variables that met normality and homogeneity of variance assumptions were presented as mean (SD), and for multiple group comparisons, one-way analysis of variance (ANOVA) was performed. Variables not meeting normality assumptions were presented as median (IQR), and the Kruskal-Wallis test was used for multiple group comparisons. Categorical variables were expressed as n (%), with group comparisons performed using the chi-square test or Fisher exact test. Postpartum BP recovery status at 6 weeks (nonrecovery=1 and recovery=0) was used as the dependent variable, and SBP, DBP, and MAP trajectory groups as independent variables. Modified Poisson regression was used to estimate risk ratios and 95% CIs for the association between gestational BP trajectories and postpartum BP nonrecovery. Sensitivity analysis was conducted among individuals with at least 4 BP measurements during pregnancy, including at least 1 per trimester, by repeating the trajectory fitting and regression analyses to assess the robustness of the main findings (Figure S2 and Table S9 in Multimedia Appendix 1).

### Ethical Considerations

This study complied with the Declaration of Helsinki and was approved by the ethics committee of Guangdong Women and Children Hospital (number 20251125). Informed consent was waived due to the retrospective study design. All data were anonymized to protect participant confidentiality. No compensation was provided to participants.

## Results

### Baseline Characteristics of Study Participants

A total of 3162 pregnant women were included in the study. Of these, 1674 were diagnosed with GH, 607 with preeclampsia, 246 with CHTN with superimposed preeclampsia, and 635 with CHTN. The proportions of women with gravidity ≥3 were 38.11% (638/1674), 42.17% (256/607), 41.87% (103/246), and 50.08% (318/635), respectively. The proportions of multiparous women were 53.11% (889/1674), 60.13% (365/607), 55.69% (137/246), and 68.35% (434/635), respectively. The median maternal age was 31.00 (27.00–35.00), 30.00 (27.00–34.00), 32.00 (28.00–36.00), and 33.00 (30.00–37.00) years, respectively. The median gestational age at delivery was 38.71 (37.57–39.71), 38.71 (37.43–39.57), 38.00 (36.71–39.11), and 38.29 (37.29–39.29) weeks, respectively (Table 1).

**Table 1:** Baseline characteristics of 3162 women with different types of hypertensive disorders of pregnancy in Western Guangdong, China, 2018-2024.

Characteristics		GH^a^ (n=1674)	Preeclampsia (n=607)	CHTN^b^ with superimposed preeclampsia (n=246)	CHTN (n=635)	*P* value	
Maternal age, median (IQR)		31.00 (27.00-35.00)	30.00 (27.00-34.00)	32.00 (28.00-36.00)	33.00 (30.00-37.00)	<.001	
**Education level, n (%)**							.06
	Above college	404 (24.13)	130 (21.42)	43 (17.48)	157 (24.72)		
	Below high school	1270 (75.87)	477 (78.58)	203 (82.52)	478 (75.28)		
**Gravidity, n (%)**							<.001
	1	633 (37.81)	187 (30.81)	81 (32.93)	151 (23.78)		
	2	403 (24.07)	164 (27.02)	62 (25.20)	166 (26.14)		
	≥3	638 (38.11)	256 (42.17)	103 (41.87)	318 (50.08)		
**Parity status, n (%)**							<.001
	Primipara	785 (46.89)	242 (39.87)	109 (44.31)	201 (31.65)		
	Multipara	889 (53.11)	365 (60.13)	137 (55.69)	434 (68.35)		
**Number of cesarean sections, n (%)**							<.001
	≥1	234 (13.98)	85 (14.00)	54 (21.95)	168 (26.46)		
	0	1440 (86.02)	522 (86.00)	192 (78.05)	467 (73.54)		
**Number of vaginal deliveries, n (%)**							.003
	≥1	698 (41.70)	297 (48.93)	98 (39.84)	299 (47.09)		
	0	976 (58.30)	310 (51.07)	148 (60.16)	336 (52.91)		
**Gynecological surgery history, n (%)**							<.001
	No	1473 (87.99)	538 (88.63)	201 (81.71)	512 (80.63)		
	Yes	201 (12.01)	69 (11.37)	45 (18.29)	123 (19.37)		
**Abortion history, n (%)**							<.001
	No	1288 (76.94)	447 (73.64)	175 (71.14)	438 (68.98)		
	Yes	386 (23.06)	160 (26.36)	71 (28.86)	197 (31.02)		
Delivery gestational age, median (IQR)		38.71 (37.57-39.71)	38.71 (37.43-39.57)	38.00 (36.71-39.11)	38.29 (37.29-39.29)	<.001	
**Mode of delivery, n (%)**							<.001
	Cesarean section	926 (55.32)	307 (50.58)	176 (71.54)	390 (61.42)		
	Vaginal delivery	748 (44.68)	300 (49.42)	70 (28.46)	245 (38.58)		
Intrapartum BMI, median (IQR)		28.08 (26.95-29.21)	28.00 (26.79-29.30)	28.38 (27.18-29.61)	28.31 (27.28-29.50)	.002	
**Neonatal sex, n (%)**							.60
	Male	900 (53.76)	318 (52.39)	137 (55.69)	355 (55.91)		
	Female	774 (46.24)	289 (47.61)	109 (44.31)	280 (44.09)		
Neonatal birth weight, median (IQR)		3000.00 (2650.00-3387.50)	3000.00 (2580.00-3400.00)	2850.00 (2270.00-3300.00)	3000.00 (2650.00-3300.00)	.001	
Apgar score at 1 minute, median (IQR)		10.00 (10.00-10.00)	10.00 (10.00-10.00)	10.00 (9.00-10.00)	10.00 (10.00-10.00)	.003	
Apgar score at 5 minutes, median (IQR)		10.00 (10.00-10.00)	10.00 (10.00-10.00)	10.00 (10.00-10.00)	10.00 (10.00-10.00)	.10	
Apgar score at 10 minutes, median (IQR)		10.00 (10.00-10.00)	10.00 (10.00-10.00)	10.00 (10.00-10.00)	10.00 (10.00-10.00)	.01	

^a^GH: gestational hypertension.

^b^CHTN: chronic hypertension.

### Trajectories of BP During Pregnancy

**Figure 1:**
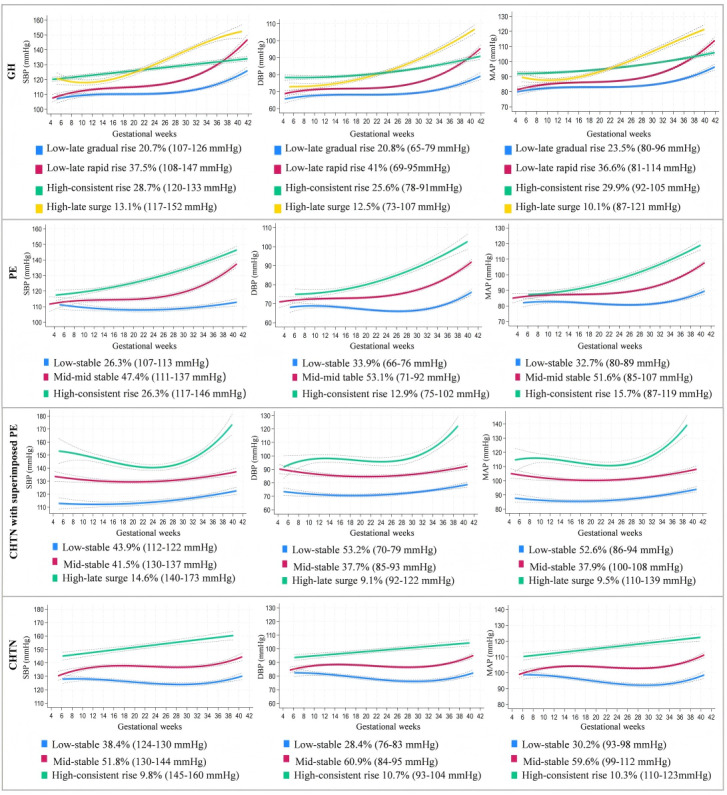
Trajectories of systolic blood pressure (SBP), diastolic blood pressure (DBP), and mean arterial pressure (MAP) during pregnancy in different types of hypertensive disorders of pregnancy in Western Guangdong, China, 2018-2024. CHTN: chronic hypertension; GH: gestational hypertension; PE: preeclampsia.

A total of 4 trajectories of SBP, DBP, and MAP were identified for GH, and 3 for preeclampsia, CHTN with superimposed preeclampsia, and CHTN. Trajectories were named according to overall relative position and slope and were consistent across SBP, DBP, and MAP within each subtype. For GH, the identified trajectories were (1) low-late gradual rise trajectory, (2) low-late rapid rise trajectory, (3) high-consistent rise trajectory, and (4) high-late surge trajectory (Figure 1). For preeclampsia, trajectories were (1) low-stable trajectory, (2) mid-mid stable trajectory, and (3) high-consistent rise trajectory (Figure 1). For CHTN with superimposed preeclampsia, trajectories were (1) low-stable trajectory, (2) mid-stable trajectory, and (3) high-late surge trajectory (Figure 1). For CHTN, the trajectories were (1) low-stable trajectory, (2) mid-stable trajectory, and (3) high-consistent rise trajectory (Figure 1).

### Incidence of Unrecovered BP at 6 Weeks Post Partum by BP Trajectories During Pregnancy

**Figure 2:**
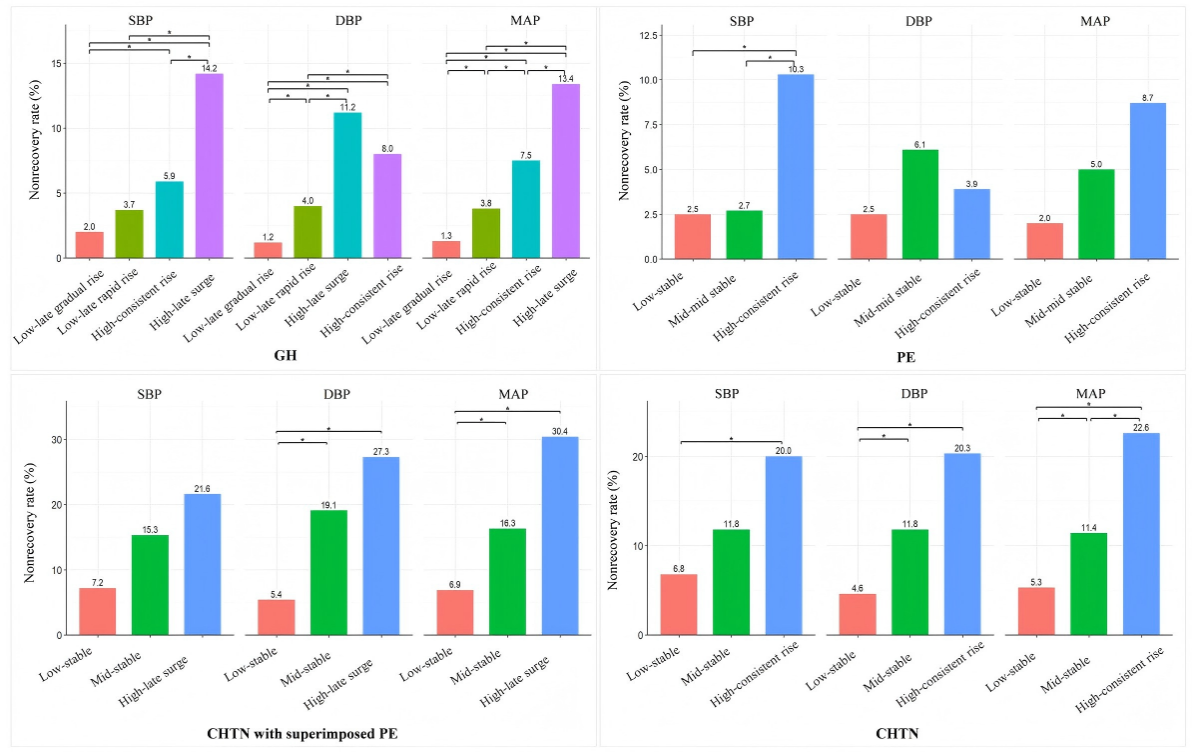
Incidence of unrecovered blood pressure (BP) at 6 weeks postpartum by BP trajectories during pregnancy in different types of hypertensive disorders of pregnancy in Western Guangdong, China, 2018-2024. CHTN: chronic hypertension; DBP: diastolic blood pressure; GH: gestational hypertension; MAP: mean arterial pressure; PE: preeclampsia; SBP: systolic blood pressure.

For GH, the high-late surge SBP trajectory was associated with the highest rate of BP nonrecovery at 6 weeks post partum (29/204, 14.2%) compared to the other 3 SBP trajectories. For DBP, there was no statistically significant difference in nonrecovery rates between the high-late surge and high-consistent rise trajectories, while significant differences were observed among the remaining DBP trajectories. For MAP, significant differences were observed among all trajectories, with the high-late surge trajectory showing the highest nonrecovery rate (22/164, 13.4%; Figure 2). For preeclampsia, only the high-consistent rise SBP trajectory had a higher rate of BP nonrecovery at 6 weeks post partum (16/155, 10.3%) than the mid-mid stable (8/293, 2.7%) and low-stable (4/159, 2.5%) SBP trajectories (Figure 2). For CHTN with superimposed preeclampsia, the high-late surge DBP trajectory had a higher rate of BP nonrecovery (6/22, 27.3%), compared to the low-stable (7/130, 5.4%) trajectory. MAP trajectories showed a similar distribution (Figure 2). For CHTN, the high-consistent rise SBP trajectory was associated with a higher rate of BP nonrecovery at 6 weeks post partum (12/60, 20%) than the low-stable trajectory (16/235, 6.8%). DBP and MAP trajectories showed a similar distribution of nonrecovery rates as the corresponding SBP trajectories (Figure 2).

### Association Between BP Trajectories During Pregnancy and the Risk of Nonrecovery to Normotension at 6 Weeks Post Partum

In the adjusted model for GH, compared to pregnant women with the low-late gradual rise SBP trajectory, those with the high-consistent rise (adjusted relative risk [aRR] 2.493, 95% CI 1.093-5.689) and high-late surge SBP trajectories (aRR 4.535, 95% CI 1.884-10.917) were associated with a significantly increased risk of BP nonrecovery at 6 weeks post partum. Similar associations were observed for MAP, with the high-consistent rise (aRR 4.838, 95% CI 1.890-12.382) and high-late surge (aRR 6.717, 95% CI 2.414-18.691) trajectories showing increased risks. For DBP trajectories, the low-late rapid rise (aRR 3.064, 95% CI 1.075-8.736), high-late surge (aRR 6.452, 95% CI 2.103-19.798), and high-consistent rise (aRR 5.603, 95% CI 1.941-16.177) trajectories were all significantly associated with increased risk of BP nonrecovery at 6 weeks post partum, compared to the low-late gradual rise trajectory (Table 2).

In the adjusted model for preeclampsia, only high-consistent rise SBP trajectory remained significantly associated with BP nonrecovery (aRR 3.355, 95% CI 1.140-9.873; Table 2).

In the adjusted model for CHTN with superimposed preeclampsia, compared to the low-stable trajectory, women with high-late surge in SBP (aRR 2.792, 95% CI 1.081-7.214), DBP (aRR 4.043, 95% CI 1.327-12.324), or MAP (aRR 4.018, 95% CI 1.462-11.045) had a significantly increased risk of BP nonrecovery at 6 weeks post partum. Additionally, women with mid-stable DBP trajectory (aRR 3.412, 95% CI 1.467-7.937) and MAP trajectory (aRR 2.280, 95% CI 1.030-5.049) were also associated with higher risk (Table 2).

**Table 2:** The association between blood pressure (BP) trajectories during pregnancy and the risk of unrecovered BP at 6 weeks among women with different types of hypertensive disorders of pregnancy (HDP) in Western Guangdong, China, 2018-2024.

HDP subtype, BP index, and trajectory group			aRR^a^ (95% CI)		
**GH^b^**					
	**SBP^c^**				
		Low-late rapid rise	1.549 (0.662-3.623)		
		High-consistent rise	2.493 (1.093-5.689)		
		High-late surge	4.535 (1.884-10.917)		
	**DBP^d^**				
		Low-late rapid rise	3.064 (1.075-8.736)		
		High-consistent rise	5.603 (1.941-16.177)		
		High-late surge	6.452 (2.103-19.798)		
	**MAP^e^**				
		Low-late rapid rise	2.558 (0.983-6.656)		
		High-consistent rise	4.838 (1.890-12.382)		
		High-late surge	6.717 (2.414-18.691)		
**Preeclampsia**					
	**SBP**				
		Mid-mid stable	0.976 (0.299-3.187)		
		High-consistent rise	3.355 (1.140-9.873)		
	**DBP**				
		Mid-mid stable	2.018 (0.758-5.373)		
		High-consistent rise	0.973 (0.227-4.180)		
	**MAP**				
		Mid-mid stable	2.144 (0.692-6.643)		
		High-consistent rise	3.445 (0.919-12.919)		
**CHTN^f^** **with superimposed preeclampsia**					
	**SBP**				
		Mid-stable	1.937 (0.826-4.543)		
		High-late surge	2.792 (1.081-7.214)		
	**DBP**				
		Mid-stable	3.412 (1.467-7.937)		
		High-late surge	4.043 (1.327-12.324)		
	**MAP**				
		Mid-stable	2.280 (1.030-5.049)		
		High-late surge	4.018 (1.462-11.045)		
**CHTN**					
	**SBP**				
		Mid-stable	1.638 (0.932-2.877)		
		High-consistent rise	2.557 (1.256-5.207)		
	**DBP**				
		Mid-stable	2.536 (1.224-5.256)		
		High-consistent rise	3.862 (1.673-8.913)		
	**MAP**				
		Mid-stable	2.095 (1.071-4.098)		
		High-consistent rise	3.714 (1.682-8.201)		

^a^aRR: adjusted relative risk.

^b^GH: gestational hypertension.

^c^SBP: systolic blood pressure.

^d^DBP: diastolic blood pressure.

^e^MAP: mean arterial pressure.

^f^CHTN: chronic hypertension.

In the adjusted model for CHTN, compared to pregnant women with the low-stable trajectory, those with the high-consistent rise trajectories of SBP (aRR 2.557, 95% CI 1.256-5.207), DBP (aRR 3.862, 95% CI 1.673-8.913), and MAP (aRR 3.714, 95% CI 1.682-8.201), as well as mid-stable trajectories of DBP (aRR 2.536, 95% CI 1.224-5.256) and MAP (aRR 2.095, 95% CI 1.071-4.098) had a significantly increased risk of BP nonrecovery at 6 weeks post partum (Table 2).

### Sensitivity Analysis

In the sensitivity analysis, 1656 individuals were diagnosed with GH, 603 with preeclampsia, 245 with CHTN with superimposed preeclampsia, and 631 with CHTN. For GH, 4 trajectories were identified for each BP type, while 3 trajectories were identified for each of the other subtypes. The changing trends of each BP trajectory were highly similar to those in the main analysis: most trajectories remained stable in early pregnancy, followed by a marked increase in the third trimester. Sustained-rising and late-acceleration BP patterns were still observed and remained statistically associated with unresolved postpartum BP (*P*<.05). For instance, regarding SBP, pregnant women who exhibited the high-consistent rise trajectories were associated with a significantly increased risk of BP nonrecovery at 6 weeks post partum (GH: aRR 2.529, 95% CI 1.110-5.765; preeclampsia: aRR 3.230, 95% CI 1.101-9.477; CHTN: aRR 2.575, 95% CI 1.262-5.250). For CHTN with superimposed preeclampsia, women with high-late surge in SBP (aRR 2.759, 95% CI 1.068-7.133) had a significantly increased risk of BP nonrecovery at 6 weeks post partum. This is similar to the results of our main analysis (Figure S2 and Table S9 in Multimedia Appendix 1).

## Discussion

### Principal Findings

In this study, GBTM were used to fit BP trajectories in pregnancy for different HDP subtypes of pregnant women. The results revealed variations in the number and shape of trajectories across HDP subtypes, which is consistent with the findings by Sinkey et al [31], who also reported divergent BP trajectories among different HDP categories [31]. Another study investigating dynamic BP changes during pregnancy similarly indicated that BP trajectories reflect, to some extent, the type of HDP, with SBP, DBP, and MAP following generally consistent overall trends within the same subtype, differing mainly in magnitude and timing [32]. In our analysis, we also found that SBP and MAP trajectories within the same subtype were highly comparable. Although SBP and DBP trends were not entirely consistent within subtypes, this finding supports the view that these 2 parameters may follow a distinct trajectory pattern [33]. Furthermore, our results demonstrated that women with HDP who exhibited higher overall BP trajectory levels during pregnancy had a greater risk of being nonrecovery to normotension at 6 weeks post partum. A prospective cohort study examining the association between early-pregnancy BP trajectories and postpartum hypertension also found a graded increase in risk for postpartum hypertension, ranging from moderate-rapid-decline to sustained-high trajectories [21]. Other studies have similarly identified risk gradients from low to high for DBP and MAP trajectories [34,35]. Within the GH subgroup, we observed that both the high-consistent rise trajectory and high-late surge trajectory for SBP, DBP, and MAP started at similar baseline levels but crossed around 22 weeks of gestation. Moreover, women with high-late surge trajectory had a higher risk of unrecovered BP at 6 weeks post partum compared to those with high-consistent rise trajectory. These findings suggest that the period around 22 weeks of gestation may represent a critical window for risk stratification and intervention. This timing aligns with the characteristic hemodynamic shift in pregnancy, during which BP normally reaches its lowest point before commencing a subsequent rise [36-38]. The divergence of pathological trajectories we identified at this juncture indicates that women who progress to GH begin to exhibit distinct deviations from the expected physiological pattern. The crossing of trajectories likely captures differing degrees of maladaptation in vascular function. Consequently, assessing BP trends across this window thus offers a dynamic metric for early risk stratification regarding postpartum BP recovery [39,40]. For women with elevated BP in the first and second trimester, intensified monitoring during the second and third trimester is recommended to facilitate timely detection of accelerating BP trends, accurate trajectory classification, and informed initiation of antihypertensive therapy. However, further research and clinical trials are warranted to validate these observations.

A systematic review showed that in women with HDP, SBP did not change significantly in the first 6 months of pregnancy and began to rise by 31 mm Hg in the third trimester [33]. Our study also identified similar patterns. Specifically, the low-late gradual rise and low-late rapid rise SBP trajectories for GH, the low-stable and mid-mid stable SBP trajectories for preeclampsia, the low-stable and mid-stable SBP trajectories for CHTN with superimposed preeclampsia, and CHTN were all relatively stable during the first and second trimesters but increased in the third trimester, with the degree of increase varying by subtype. Additionally, across all 4 HDP subtypes, we identified a subset of women with specific SBP trajectories characterized by high baseline levels and SBP values exceeding 140 mm Hg in the third trimester. Although the high-consistent rise trajectory in GH did not exceed this threshold in the third trimester, it had the highest baseline level and showed a linear rising trend. Our results indicate that these women had greater difficulty achieving BP recovery at 6 weeks post partum, consistent with previous studies reporting that more severe and longer duration of HDP are associated with greater vascular, renal, and multisystem injury, leading to poorer postpartum BP recovery [8,18]. In this study, SBP trajectories across all 4 HDP types were significantly associated with the risk of unrecovered BP at 6 weeks post partum. This may be attributable to SBP being a better predictor of CVD [41], providing biological plausibility for our findings.

Regarding DBP trends, our study showed different trends, mainly in the second trimester. Specifically, women with GH, preeclampsia, and CHTN who exhibited high-consistent rise trajectories, as well as those with GH showing high-late surge trajectory, all demonstrated rising DBP during the second trimester. This is similar to previous research reports stating that in women with HDP, DBP shows an upward trend, increasing by approximately 5 mm Hg in the second trimester compared with the first trimester [42,43]. Additionally, some trajectories in our study—such as the low-late gradual rise and low-late rapid rise trajectories in GH, and the low-stable and mid-mid stable trajectories in preeclampsia, as well as the low-stable and mid-stable trajectories in CHTN—did not show significant rising or falling trends during the second trimester but began to increase toward the end of the second trimester. This trend differs from most previous reports. Similar trends were observed in CHTN with superimposed preeclampsia. Notably, previous studies have suggested that women with GH may experience a distinct time point—occurring at various stages of pregnancy—at which DBP rises abruptly. Before this point, DBP typically remains stable and within the normal range, but increases sharply thereafter [44], further supporting the concept of heterogeneous dynamic BP changes during pregnancy. Finally, across all subtypes, DBP trajectories significantly associated with unrecovered BP at 6 weeks post partum (*P*<.05) were characterized by higher overall levels. Specifically, in women with CHTN with superimposed preeclampsia and those with CHTN, the relevant trajectories reached DBP ≥90 mm Hg before 20 weeks of gestation, whereas in women with GH, this threshold was attained after 20 weeks.

In this study, baseline levels and trends of MAP were similar to those of SBP. Similar to the SBP results, pregnant women with GH or CHTN who exhibited high-consistent rise MAP trajectory, as well as those with GH and CHTN with superimposed preeclampsia who exhibited high-late surge MAP trajectory, were all at high risk for unrecovered BP at 6 weeks post partum. Unlike the SBP trajectories, the mid-stable MAP trajectory in the CHTN with superimposed preeclampsia and the CHTN remained statistically associated with the risk of unrecovered BP at 6 weeks post partum. Previous studies have shown that MAP has predictive value for adverse pregnancy outcomes [45] and may be superior to both SBP and DBP in clinical application [46], possibly because elevated MAP induces abnormalities in the nitric oxide (NO) synthesis process, thereby triggering a series of vascular dysfunctions [47]. However, research on the application of MAP in pregnancy is still limited [48], and further studies are needed.

Giorgione et al [49] demonstrated that persistent postpartum hypertension following HDP is predicted by a confluence of demographic factors (age or Afro-Caribbean ethnicity), clinical metrics (early pregnancy MAP and BMI), and, most significantly, peripartum echocardiographic evidence of pathological cardiac remodeling (elevated left ventricular mass index or relative wall thickness) and subclinical myocardial dysfunction (impaired global longitudinal strain and diastolic indices). Their findings posit that persistent hypertension reflects underlying cardiovascular maladaptations unmasked by pregnancy, which can be effectively identified through peripartum screening [49]. Little is known about the potential mechanisms linking dynamic changes in BP during pregnancy to postpartum BP recovery status. A study using a new 3D miniheart model revealed that GH may activate cell death signaling pathways and promote cardiac fibrosis, leading to cardiovascular damage; preeclampsia may cause vascular dysfunction-related inflammation, protein disorders, and endothelial dysfunction, thereby adversely affecting cardiovascular health [50]. A retrospective analysis of patients with preeclampsia and CHTN with superimposed preeclampsia demonstrated that autonomic dysregulation may occur within the first week post partum [51]. An imbalance between sympathetic and parasympathetic regulation can promote elevated BP and peripheral vasoconstriction, thereby increasing cardiac afterload and myocardial oxygen consumption [51]. Moreover, women with CHTN, who typically have hypertension prior to pregnancy, often present with underlying endothelial dysfunction. This dysfunction may be further aggravated by systemic inflammation during pregnancy, contributing to persistent postpartum hypertension [52].

Endothelial dysfunction is an early pathological change in hypertension [53,54]. Soluble fms-like tyrosine kinase-1 (sFlt1) and placental growth factor (PLGF) are important factors affecting maternal endothelial function, and their abnormal expression is closely related to endothelial dysfunction. sFlt1 is an antiangiogenic soluble protein, while PLGF promotes placental angiogenesis and repair [55,56]. Studies have shown that increased circulating sFlt1 and decreased proangiogenic factors such as PLGF contribute to impaired placental angiogenesis [57]. The imbalance of angiogenic factors can lead to maternal systemic endothelial dysfunction and vascular hyperreactivity, and clinical symptoms such as hypertension, proteinuria, and organ dysfunction [58]. One study showed that serum PLGF levels in the HDP group were significantly lower than those in the normal pregnancy group and declined further with increasing disease severity. Specifically, PLGF levels showed a significant stepwise decline from GH to mild preeclampsia and severe preeclampsia [59]. NO is a key endogenous molecule for regulating vascular tone and maintaining BP stability [60]. Studies indicate that plasma endothelial NO synthase levels are significantly lower in women with GH and preeclampsia compared to normal pregnant women, with the lowest levels in the preeclampsia group. Reduced endothelial NO synthase leads to impaired NO synthesis [61]. Decreased NO bioavailability results in vascular relaxation disorders, increased systemic vascular resistance, elevated BP, and impaired placental and fetal development [62]. In addition, elevated asymmetric dimethylarginine, reduced dimethylarginine dimethylaminohydrolases, and insufficient L-arginine transport in patients with preeclampsia also inhibit NO production [62].

Based on these mechanisms and the trajectory disparities identified in our study, we recommend stratified management for women with HDP. For those with sustained‑rise or late‑acceleration trajectories, postpartum BP often remains difficult to normalize. These high-risk patients can be classified as key populations for monitoring during pregnancy, with daily BP measurements at fixed times, the plotting of BP trend charts, clarification of the type of hypertension, and early implementation of pharmacological interventions. These patients should also receive prioritized and more frequent postpartum follow-up visits, with prolongation of the follow-up duration, in order to reduce their long-term risk of CVD [63-65]. For pregnant women with low-risk BP trajectories, nonpharmacological interventions should also be emphasized [48]. A meta-analysis demonstrated that moderate walking, upper and lower limb muscles training, and stretching exercises are more effective in reducing gestational SBP and DBP in women with HDP than healthy pregnant women [66]. Deep breathing exercise and progressive muscular relaxation can significantly reduce SBP, DBP, and MAP in pregnant women with mild preeclampsia [67,68]. Therefore, a combination of pharmacological and nonpharmacological approaches tailored to BP trajectory type during pregnancy may help manage BP and reduce the risk of postpartum BP not returning to normal.

Postpartum follow-up guidelines for patients with HDP are lacking or insufficient in most countries [69]. Early diagnosis, risk stratification, standardized postpartum care, and strict BP management can effectively reduce postpartum cardiovascular risk and mortality [64]. Our findings indicate that BP trajectories during pregnancy are valuable for HDP risk stratification and for identifying women at high risk of poor postpartum BP recovery. Incorporating BP trajectories into postpartum follow-up may optimize management and improve both short- and long-term cardiovascular outcomes. Currently, predictive models based on BP trajectories during pregnancy are in the stages of algorithm validation and prospective testing [70]. Trajectories are constructed from BP data collected under routine clinical conditions, demonstrating practicality and feasibility. Emerging evidence suggests that the early postpartum period represents a critical therapeutic window for mitigating the long-term cardiovascular sequelae of HDP. For instance, a self-management intervention optimizing BP control within the first few weeks post partum led to favorable left ventricular and left atrial remodeling that was evident nearly a year later [13].

Developing a trajectory-specific tool to predict postpartum BP nonrecovery could enhance risk assessment and facilitate personalized intervention and follow-up strategies. It should be noted that postpartum BP recovery status is also influenced by multiple factors, such as maternal serum albumin levels, family history of hypertension, and prepregnancy body weight [18]. Further studies should explore the interactions between these risk factors and BP trajectories to better elucidate the relationship between BP dynamics during pregnancy and postpartum BP recovery status.

This study used real-world data from women with HDP, all of whom had at least 3 BP measurements during pregnancy spanning the first, second, and third trimesters. This comprehensive sampling strengthens the reliability of the findings. Sensitivity analyses further supported the robustness of our results: the number, trends, and relative proportion of trajectory groups in each subtype were generally consistent with those in the main analysis, with only minor changes in statistical associations. To enhance internal validity, we excluded multiple pregnancies, as this condition represents an important cardiovascular risk factor that may confound the association between BP trajectories and postpartum outcomes.

Nevertheless, this study has several limitations. First, we lacked data on antihypertensive use, so we could not adjust for potential confounding by treatment. While this may have led to some underestimation of true BP levels, the associations held across HDP subtypes—including in CHTN, where treatment is most likely—and remained consistent in direction and magnitude. Because antihypertensive therapy is part of routine care, the trajectories observed here reflect real-world BP patterns under standard clinical management. Second, owing to follow-up challenges, only BP recovery at 6 weeks post partum was assessed; longer-term BP recovery status remains unexplored. Future large-scale, multicenter cohort studies with extended follow-up are therefore warranted to further validate these findings. This study suggests a strong association between antenatal BP trajectories during pregnancy and failure to achieve normotension by 6 weeks post partum. As an observational study, however, our results indicate correlation rather than causation. These findings contribute to the understanding of postpartum BP changes in women with HDP, but further prospective and mechanistic studies are needed for validation.

### Conclusion

This study identified distinct antepartum BP trajectories across HDP subtypes, with MAP and SBP showing similar trends and DBP showing more variability. Sustained-rising and late-acceleration patterns, particularly in GH, CHTN with superimposed preeclampsia, and CHTN, were associated with a higher risk of nonrecovery to normotension at 6 weeks post partum. In preeclampsia, only the high-consistent rise SBP trajectory predicted nonrecovery of normotension postpartum. Trajectory analysis, therefore, could support early risk identification and individualized postpartum care to improve long-term cardiovascular outcomes in patients with HDP.

## Appendix

Multimedia Appendix 1 Supplementary figures and tables.

## Abbreviations

aRR: adjusted relative risk
BP: blood pressure
CHTN: chronic hypertension
CVD: cardiovascular disease
DBP: diastolic blood pressure
GBTM: group-based trajectory modeling
GH: gestational hypertension
HDP: hypertensive disorders of pregnancy
MAP: mean arterial pressure
NO: nitric oxide
PLGF: placental growth factor
SBP: systolic blood pressure
sFlt1: soluble fms-like tyrosine kinase-1

## Data Availability

The data that support the findings of this study are available from the corresponding author upon reasonable request.
