# Supplementary material for: Association Between Gestational Blood Pressure Trajectories and Postpartum Normotension Recovery in Hypertensive Disorders: Retrospective Cohort Study
Source: JMIR Public Health Surveill. 2026 Apr 30;12:e89295. doi: 10.2196/89295 (PMC13176814; doi:10.2196/89295)
Supplement: Multimedia Appendix 1 [file publichealth_v12i1e89295_app1.docx]

**
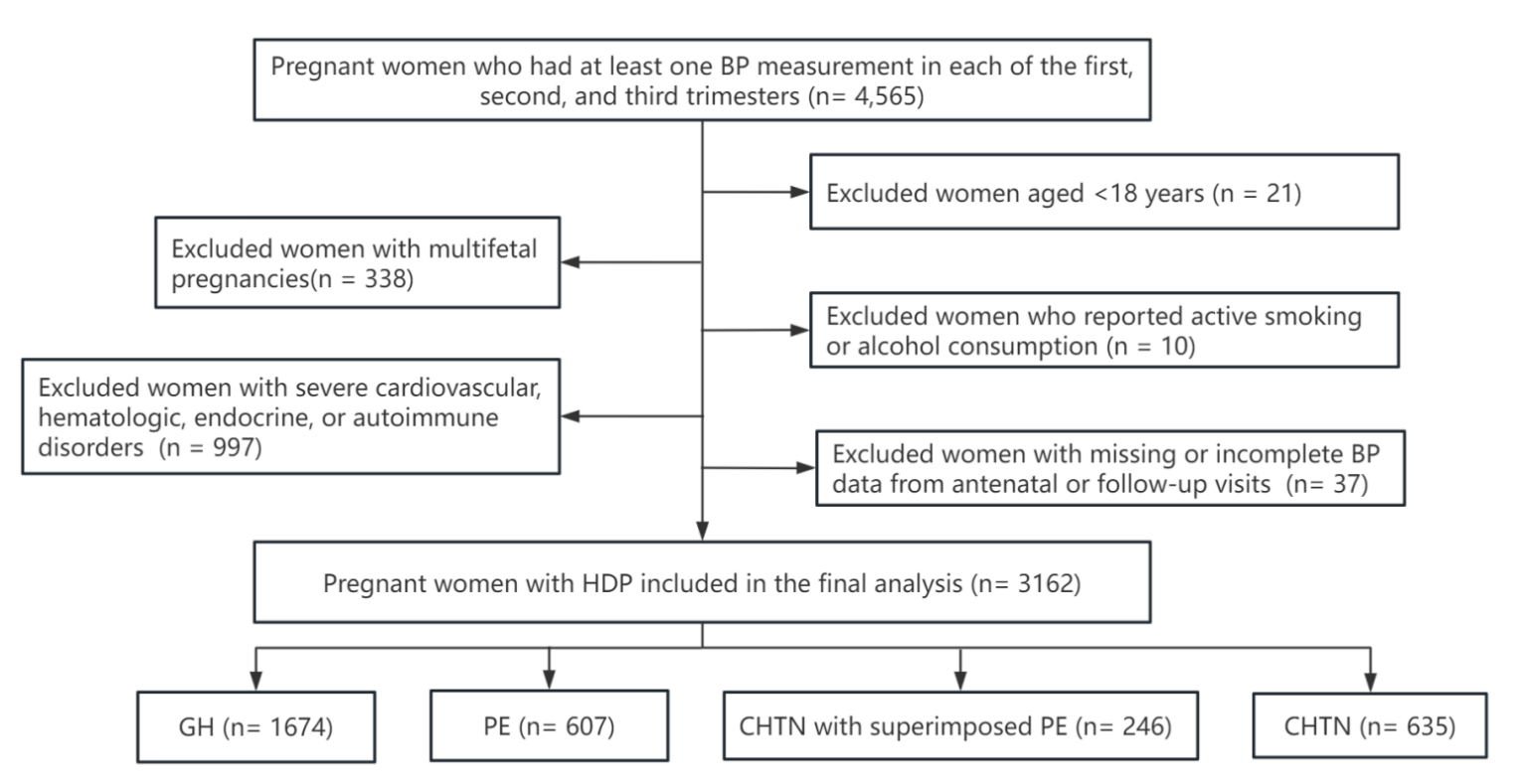
**

**Figure S1.** Flow chart for selection process of the study.

**Abbreviations:** BP, blood pressure; HDP, hypertensive disorders in pregnancy; GH, gestational hypertension; PE, preeclampsia; CHTN with superimposed PE, chronic hypertension with superimposed preeclampsia; CHTN, chronic hypertension.

**Table S1.** Model selection Indicators in the fitting process of GBTM for women with GH.

| BP | Group | Order | AIC | BIC | Proportions per class (%) | AvePP | Pj(%) | πj(%) | occ |
| --- | --- | --- | --- | --- | --- | --- | --- | --- | --- |
| SBP | 2 | 32 | -48150.28 | -48174.68 | 49.5/50.5 | 0.92 / 0.92 | 49.47/50.53 | 49.34/50.66 | 11.14/10.51 |
|  | 3 | 333 | -47915.12 | -47955.79 | 32.6/53.7/13.7 | 0.89 / 0.87 / 0.86 | 32.60/53.73/13.67 | 31.96/55.73/12.31 | 6.88/5.29/44.50 |
|  | 4 | 3313 | -47749.46 | -47798.26 | 20.74/37.49/28.68/13.09 | 0.85 / 0.79 / 0.80 / 0.83 | 20.74/37.49/28.68/13.09 | 20.49/39.13/28.20/12.19 | 22.82/5.75/9.94/35.47 |
|  | 5 | 22212 | -47728.81 | -47780.33 | 19.25/37.88/15.37/26.58/0.92 | 0.85 / 0.79 / 0.78 / 078 / 0.95 | 19.25/37.88/15.37/26.58/0.92 | 18.64/39.31/15.35/25.93/0.78 | 26.48/5.96/19.01/10.01/2247.04 |
| DBP | 2 | 32 | -45066.81 | -45091.22 | 48.82/51.18 | 0.92 / 0.92 | 48.82/51.18 | 48.98/51.02 | 11.40/11.49 |
|  | 3 | 332 | -44841.69 | -44879.65 | 21.06/47.89/31.05 | 0.84 / 0.84 / 0.87 | 21.06/47.89/31.05 | 20.67/48.75/30.59 | 20.58/5.40/15.78 |
|  | 4 | 3322 | -44718.21 | -44767.01 | 20.79/41.02/12.55/25.65 | 0.85 / 0.80 / 0.81 / 0.79 | 20.79/41.02/12.55/25.65 | 20.43/43.01/11.77/24.79 | 21.74/5.40/31.95/11.79 |
|  | 5 | 33333 | -44626.81 | -44694.60 | 13.52/16.31/25.23/39.17/5.77 | 0.85 / 0.71 / 0.80 / 0.80 / 0.80 | 13.52/16.31/25.23/39.17/5.77 | 12.31/15.29/24.67/42.11/5.62 | 39.40/14.02/12.48/5.56/69.02 |
|  | 6 | 332233 | -44571.45 | -44647.37 | 13.71/21.16/22.39/21.31/4.68/16.75 | 0.82 / 0.69 / 0.68 / 0.78 / 0.81 / 0.70 | 13.71/21.16/22.39/21.31/4.68/16.75 | 13.62/21.51/21.88/21.57/4.55/17.08 | 28.16/8.23/7.86/12.57/90.51/11.12 |
| MAP | 2 | 32 | -45088.86 | -45113.26 | 47.97/52.03 | 0.92 / 0.93 | 47.97/52.03 | 48.15/51.85 | 12.27/12.08 |
|  | 3 | 333 | -44847.12 | -44887.79 | 26.27/50.73/22.99 | 0.89 / 0.84 / 0.85 | 26.27/50.73/22.99 | 25.09/52.99/21.92 | 23.01/4.72/20.48 |
|  | 4 | 3323 | -44675.57 | -44727.08 | 23.48/36.56/10.09/29.87 | 0.87 / 0.79 / 0.82 / 0.83 | 23.44/36.45/29.92/10.19 | 22.94/37.69/29.51/9.86 | 22.15/6.26/11.25/44.94 |
|  | 5 | 33233 | -44559.19 | -44624.27 | 15.04/15.72/27.16/36.22/5.86 | 0.84 / 0.74 / 0.82 / 0.80 / 0.85 | 15.04/15.72/27.16/36.22/5.86 | 14.64/14.28/27.36/38.11/5.62 | 30.47/17.84/12.48/6.40/97.73 |
|  | 6 | 333323 | -44502.22 | -44580.85 | 15.56/14.70/21.95/21.18/21.32/5.30 | 0.83 / 0.72 / 0.71 / 0.78 / 0.70 / 0.86 | 15.56/14.70/21.95/21.18/21.32/5.30 | 15.83/14.76/22.28/22.10/20.01/5.02 | 26.13/14.90/8.69/12.29/9.14/116.52 |
|  | 7 | 3332232 | -44454.64 | -44541.41 | 15.81/14.94/18.18/5.78/21.28/23.68/0.35 | 0.71 / 0.81 / 0.69 / 0.82 / 0.77 / 0.70 / 0.97 | 15.81/14.94/18.18/5.78/21.28/23.68/0.35 | 15.65/15.47/17.44/5.68/22.46/22.94/0.36 | 13.82/24.43/11.00/78.56/12.14/8.15/7819.96 |

**Note:** Order, polynomial order for each trajectory

**Abbreviations:** GBTM, Group-based trajectory modeling; BP, blood pressure; SBP, systolic blood pressure; DBP, diastolic blood pressure；MAP, mean arterial pressure; GH, gestational hypertension; AIC, Akaike information criterion; BIC, Bayesian information criterion; AvePP, average posterior probability of assignment; Pj, the posterior probability of group membership; πj, the actual proportion of group membership; OCC, odds of correct classification.

.**Table S2.** Model selection Indicators in the fitting process of GBTM for women with PE.

| BP | Group | Order | AIC | BIC | Proportions per class (%) | AvePP | Pj(%) | | πj(%) | occ |
| --- | --- | --- | --- | --- | --- | --- | --- | --- | --- | --- |
| SBP | 2 | 33 | -18350.44 | -18372.48 | 48.56/51.44 | 0.94 / 0.94 | 48.56/51.44 | | 48.43/51.57 | 15.34/13.87 |
|  | 3 | 232 | -18182.39 | -18211.05 | 26.31/47.37/26.32 | 0.90 / 0.88 / 0.91 | 26.31/47.37/26.32 | | 26.19/48.27/25.54 | 24.37/7.63/28.97 |
|  | 4 | 2332 | -18083.16 | -18122.84 | 23.98/41.14/12.88/21.99 | 0.90 / 0.87 / 0.85 / 0.87 | 23.98/41.14/12.88/21.99 | | 23.56/41.85/12.69/21.91 | 28.34/9.10/40.35/23.85 |
|  | 5 | 23213 | -18007.22 | -18053.51 | 22.18/36.42/7.89/20.75/12.77 | 0.89 / 0.85 / 0.85 / 0.86 / 0.84 | 22.18/37.23/8.24/19.77/12.77 | | 22.24/37.23/8.24/19.77/12.77 | 27.03/9.25/64.40/25.86/36.70 |
|  | 6 | 232222 | -17980.91 | -18036.02 | 8.24/10.07/23.28/9.61/24.35/24.46 | 0.86 / 0.86 / 0.83 / 0.87 / 0.80 / 0.86 | 6.01/11.62/26.04/6.12/33.18/19.41 | | 4.94/10.87/26.04/6.10/33.11/18.95 | 121.96/50.63/14/106.15/8.14/26.44 |
|  | 7 | 2222222 | -17955.07 | -18016.78 | 6.49/9.95/23.86/4.40/18.74/13.19/23.36 | 0.82 / 0.84 / 0.81 / 0.89 / 0.77 / 0.85 / 0.78 | | 6.49/9.95/23.86/4.40/18.74/13.19/23.36 | 6.26/10.21/25.21/4.61/16.97/12.52/24.22 | 66.62/47.69/12.42/171.75/16.39/39.91/11.14 |
| DBP | 2 | 32 | -17039.53 | -17059.37 | 44.35/55.65 | 0.93 / 0.95 | 44.35/55.65 | | 45.14/54.86 | 15.10/16.70 |
|  | 3 | 332 | -16878.05 | -16908.91 | 33.93/53.12/12.95 | 0.93 / 0.91 / 0.87 | 33.93/53.12/12.95 | | 33.11/54.20/12.69 | 28.56/8.47/45.96 |
|  | 4 | 3322 | -16801.99 | -16841.67 | 34.64/33.66/24.63/7.07 | 0.92 / 0.82 / 0.84 / 0.84 | 34.64/33.66/24.63/7.07 | | 35.26/34.66/22.90/7.25 | 21.59/8.71/17.83/68.25 |
|  | 5 | 33322 | -16728.60 | -16779.30 | 24.69/15.71/29.42/24.81/5.36 | 0.89 / 0.81 / 0.81 / 0.85 / 0.88 | 24.69/15.71/29.42/24.81/5.36 | | 24.71/15.82/29.00/25.21/5.27 | 23.53/22.11/10.14/16.81/135.77 |
|  | 6 | 333321 | -16698.03 | -16757.54 | 24.12/15.34/3.18/29.11/25.04/3.23 | 0.88 / 0.78 / 0.85 / 0.80 / 0.86 / 0.91 | 24.12/15.34/3.18/29.11/25.04/3.23 | | 24.22/14.83/2.97/29.49/25.37/3.13 | 23.60/20.96/187.83/9.43/18.03/318.80 |
| MAP | 2 | 32 | -17135.27 | -17155.10 | 44.74/55.26 | 0.95 / 0.95 | 44.74/55.26 | | 44.48/55.52 | 22.47/16.29 |
|  | 3 | 332 | -16954.74 | -16985.60 | 32.71/51.63/15.66 | 0.93 / 0.91 / 0.89 | 32.71/51.63/15.66 | | 32.62/52.22/15.16 | 25.69/9.39/47.09 |
|  | 4 | 3322 | -16859.42 | -16899.10 | 30.33/31.53/26.67/11.47 | 0.82 / 0.93 / 0.84 / 0.87 | 30.33/31.53/26.67/11.47 | | 30.31/31.63/26.69/11.37 | 10.49/27.35/14.62/51.18 |
|  | 5 | 33322 | -16752.22 | -16802.92 | 22.16/13.33/33.05/25.00/6.46 | 0.89 / 0.85 / 0.85 / 0.84 / 0.91 | 22.16/13.33/33.05/25.00/6.46 | | 22.41/13.01/32.13/26.19/6.26 | 27.36/38.68/12.27/15.49/146.95 |
|  | 6 | 233222 | -16728.85 | -16786.16 | 24.63/11.96/23.02/22.21/13.07/5.10 | 0.89 / 0.86 / 0.79 / 0.76 / 0.82 / 0.91 | 24.63/11.96/23.02/22.21/13.07/5.10 | | 25.21/11.53/22.57/23.56/12.03/5.11 | 24.02/50.95/12.95/10.40/32.99/188.20 |
|  | 7 | 2222222 | -16707.66 | -16769.38 | 6.65/11.46/25.19/17.11/10.63/23.99/4.98 | 0.86 / 0.83 / 0.83 / 0.81/0.84 / 0.82 / 0.88 | 6.65/11.46/25.19/17.11/10.63/23.99/4.98 | | 6.26/11.86/27.02/15.32/9.39/24.88/5.27 | 91.60/35.73/13.20/23.71/50.85/13.31/133.82 |

**Note:** Order, polynomial order for each trajectory

**Abbreviations:** GBTM, Group-based trajectory modeling; BP, blood pressure; SBP, systolic blood pressure; DBP, diastolic blood pressure；MAP, mean arterial pressure; PE, preeclampsia; AIC, Akaike information criterion; BIC, Bayesian information criterion; AvePP, average posterior probability of assignment; Pj, the posterior probability of group membership; πj, the actual proportion of group membership; OCC, odds of correct classification.

**Table S3.** Model selection Indicators in the fitting process of GBTM for women with CHTN with superimposed PE.

| BP | Group | Order | AIC | BIC | Proportions per class (%) | AvePP | Pj(%) | πj(%) | occ |
| --- | --- | --- | --- | --- | --- | --- | --- | --- | --- |
| SBP | 2 | 22 | -7662.03 | -7676.06 | 56.17/43.83 | 0.97 / 0.96 | 56.17/43.83 | 56.50/43.50 | 21.46/33.56 |
|  | 3 | 223 | -7551.28 | -7574.07 | 43.88/41.51/14.65 | 0.45 / 0.40 / 0.15 | 43.88/41.51/14.60 | 45.12/39.84/15.04 | 20.02/29.28/75.55 |
|  | 4 | 3223 | -7519.91 | -7551.46 | 31.77/30.16/28.34/9.73 | 0.92 / 0.85 / 0.87 / 0.86 / 0.94 | 31.77/30.16/28.34/9.73 | 32.11/29.27/29.67/8.94 | 23.30/13.42/15.19/165.00 |
|  | 5 | 12223 | -7497.27 | -7532.32 | 30.87/30.04/10.47/22.81/5.80 | 0.91 / 0.86 / 0.86 / 0.82 / 0.94 | 30.87/30.04/10.47/22.81/5.80 | 31.30/28.86/9.76/23.98/6.10 | 23.57/14.88/59.46/14.70/248.15 |
| DBP | 2 | 22 | -7179.05 | -7193.07 | 61.14/38.86 | 0.97 / 0.97 | 61.14/38.86 | 61.79/38.21 | 21.47/55.53 |
|  | 3 | 223 | -7079.36 | -7102.14 | 53.18/37.73/9.10 | 0.97 / 0.93 / 0.94 | 53.18/37.73/9.10 | 52.85/38.21/8.94 | 26.67/20.87/154.38 |
|  | 4 | 3233 | -7042.67 | -7075.97 | 42.26/27.47/23.63/6.64 | 0.92 / 0.84 / 0.92 / 0.94 | 42.26/27.47/23.63/6.64 | 44.31/26.83/23.36/6.50 | 14.22/14.28/37.51/213.91 |
|  | 5 | 22231 | -7034.15 | -7069.21 | 22.97/30.88/23.06/17.65/5.45 | 0.83 / 0.80 / 0.83 / 0.89 / 0.89 | 22.97/30.88/23.06/17.65/5.45 | 23.58/31.30/22.36/17.07/5.69 | 15.56/8.62/17.44/38.84/140.04 |
| MAP | 2 | 22 | -7219.88 | -7233.90 | 58.82/41.18 | 0.97 / 0.97 | 58.82/41.18 | 58.94/41.06 | 26.69/41.26 |
|  | 3 | 223 | -7106.68 | -7129.46 | 52.62/37.93/9.45 | 0.96 / 0.95 / 0.97 | 52.62/37.93/9.45 | 53.25/37.40/9.35 | 23.42/35.07/281.04 |
|  | 4 | 2233 | -7055.07 | -7086.62 | 33.39/28.66/29.34/8.61 | 0.92 / 0.88 / 0.95 / 0.94 | 33.39/28.66/29.34/8.61 | 34.55/28.05/28.46/8.94 | 22.37/19.58/48.72/152.34 |
|  | 5 | 22231 | -7016.70 | -7051.75 | 33.00/28.32/28.35/3.17/7.16 | 0.92 / 0.89 / 0.95 / 0.95 / 0.98 | 33.00/28.32/28.35/3.17/7.16 | 34.55/27.64/27.64/3.26/6.91 | 21.16/20.37/50.37/581.46/812.68 |

**Note:** Order, polynomial order for each trajectory

**Abbreviations:** GBTM, Group-based trajectory modeling; CHTN with superimposed PE, chronic hypertension with superimposed preeclampsia; BP, blood pressure; SBP, systolic blood

pressure; DBP, diastolic blood pressure；MAP, mean arterial pressure; AIC, Akaike information criterion; BIC, Bayesian information criterion; AvePP, average posterior probability of

assignment; Pj, the posterior probability of group membership; πj, the actual proportion of group membership; OCC, odds of correct classification.

**Table S4.** Model selection Indicators in the fitting process of GBTM for women with CHTN.

| BP | Group | Order | AIC | BIC | Proportions per class (%) | AvePP | Pj(%) | πj(%) | occ |
| --- | --- | --- | --- | --- | --- | --- | --- | --- | --- |
| SBP | 2 | 33 | -20571.99 | -20594.25 | 64.15/35.85 | 0.94 / 0.92 | 64.15/35.85 | 65.83/34.17 | 7.47/23.37 |
|  | 3 | 331 | -20416.84 | -20458.79 | 38.48/51.83/9.76 | 0.90 / 0.88 / 0.90 | 38.40/51.84/9.76 | 37.01/53.54/9.45 | 16.00/6.58/83.85 |
|  | 4 | 2331 | -20363.82 | -20401.67 | 5.66/39.90/47.48/6.96 | 0.89 / 0.86 / 0.88 / 0.90 | 5.67/39.90/47.48/6.97 | 5.51/40.16/47.87/6.46 | 134.14/9.10/8.33/132.46 |
|  | 5 | 23331 | -20311.97 | -20360.96 | 5.59/43.93/8.65/35.03/6.80 | 0.90 / 0.88 / 0.82 / 0.84 / 0.87 | 5.60/43.90/8.65/35.03/6.80 | 5.35/44.57/8.19/34.80/7.09 | 157.60/8.99/51.66/9.72/88.19 |
| DBP | 2 | 44 | -19156.00 | -19182.72 | 49.70/50.30 | 0.93 / 0.91 | 49.70/50.30 | 49.86/50.14 | 13.41/9.66 |
|  | 3 | 331 | -19028.39 | -19057.34 | 28.39/60.88/10.73 | 0.89 / 0.91 / 0.89 | 28.39/60.89/10.08 | 27.40/62.52/10.08 | 21.37/5.94/74.82 |
|  | 4 | 2331 | -18956.22 | -18994.08 | 9.48/42.12/41.29/7.11 | 0.85 / 0.85 / 0.85 / 0.89 | 9.48/42.13/41.29/7.11 | 9.45/43.62/40.31/6.66 | 53.29/7.21/8.65/117.89 |
|  | 5 | 23333 | 18920.09 | -18973.54 | 9.68/12.33/31.16/40.73/6.10 | 0.86 / 0.73 / 0.78 / 0.85 / 0.92 | 9.70/12.33/31.16/40.73/6.10 | 9.61/10.71/33.07/41.10/5.51 | 57.37/22.76/7.17/8.20/202.90 |
|  | 6 | 243432 | -18899.51 | -18966.32 | 7.17/18.89/15.55/33.72/19.46/5.20 | 0.82 / 0.71 / 0.69 / 0.73 / 0.73 / 0.90 | 7.17/18.89/15.55/33.72/19.46/5.20 | 6.93/19.21/14.65/35.59/18.43/5.20 | 60.60/10.51/13.26/4.96/12.20/162.29 |
| MAP | 2 | 33 | 19273.06 | -19295.33 | 54.54/45.46 | 0.94 / 0.91 | 54.54/45.46 | 53.23/46.77 | 14.38/11.02 |
|  | 3 | 331 | -19098.03 | -19126.98 | 30.18/59.55/10.27 | 0.90 / 0.92 / 0.93 | 30.18/59.55/10.27 | 29.45/60.79/9.76 | 20.69/7.23/121.42 |
|  | 4 | 2331 | -19030.58 | -19068.43 | 9.38/40.82/41.56/8.24 | 0.85 / 0.86 / 0.87 / 0.89 | 9.38/40.82/41.56/8.24 | 9.13/40.47/42.05/8.35 | 54.41/8.82/9.09/88.58 |
|  | 5 | 23331 | -18979.28 | -19028.27 | 9.50/37.71/21.91/22.75/8.14 | 0.85 / 0.85 / 0.75 / 0.77 / 0.88 | 9.50/37.71/21.91/22.75/8.14 | 9.45/37.95/21.57/22.52/8.50 | 54.81/9.32/11.14/11.52/78.69 |
|  | 6 | 233331 | -19018.05 | -18957.92 | 8.92/22.76/5.64/31.25/24.42/7.02 | 0.84 / 0.70 / 0.77 / 0.80 / 0.79 / 0.88 | 8.92/22.76/5.64/31.25/24.42/7.02 | 9.13/21.42/5.83/32.28/24.25/7.09 | 52.04/8.68/54.40/8.51/11.90/97.13 |

**Note:** Order, polynomial order for each trajectory

**Abbreviations:** GBTM, Group-based trajectory modeling; CHTN, chronic hypertension; BP, blood pressure; SBP, systolic blood pressure; DBP, diastolic blood pressure; MAP, mean

arterial pressure; AIC, Akaike information criterion; BIC, Bayesian information criterion; AvePP, average posterior probability of assignment; Pj, the posterior probability of group

membership; πj, the actual proportion of group membership; OCC, odds of correct classification.

**Table S5.** Model fitting parameters for BP trajectories during pregnancy in women with GH.

| BP | Trajectory name | Parameter | Estimate | Standard Error | T for H_0_:Parameter=0 | Prob > \|T\| |
| --- | --- | --- | --- | --- | --- | --- |
| SBP | Low-late gradual rise | Intercept | 101.95712 | 3.37238 | 30.233 | 0.0000 |
|  |  | Linear | 1.31445 | 0.50187 | 2.619 | 0.0088 |
|  |  | Quadratic | -0.07013 | 0.02243 | -3.126 | 0.0018 |
|  |  | Cubic | 0.00125 | 0.00031 | 4.043 | 0.0001 |
|  | Low-late rapid rise | Intercept | 100.41775 | 2.56182 | 39.198 | 0.0000 |
|  |  | Linear | 2.06344 | 0.37965 | 5.435 | 0.0000 |
|  |  | Quadratic | -0.10709 | 0.01751 | -6.115 | 0.0000 |
|  |  | Cubic | 0.00201 | 0.00025 | 7.911 | 0.0000 |
|  | High-consistent rise | Intercept | 118.47698 | 0.77481 | 152.910 | 0.0000 |
|  |  | Linear | 0.36994 | 0.02729 | 13.554 | 0.0000 |
|  | High-late Surge | Intercept | 129.93581 | 4.54869 | 28.566 | 0.0000 |
|  |  | Linear | -2.48395 | 0.69664 | -3.566 | 0.0004 |
|  |  | Quadratic | 0.14573 | 0.03236 | 4.504 | 0.0000 |
|  |  | Cubic | -0.00175 | 0.00046 | -3.773 | 0.0002 |
| DBP | Low-late gradual rise | Intercept | 62.50872 | 2.44446 | 25.572 | 0.0000 |
|  |  | Linear | 0.92960 | 0.37138 | 2.503 | 0.0123 |
|  |  | Quadratic | -0.05034 | 0.01689 | -2.981 | 0.0029 |
|  |  | Cubic | 0.00090 | 0.00024 | 3.795 | 0.0001 |
|  | Low-late rapid rise | Intercept | 64.89957 | 1.86062 | 34.881 | 0.0000 |
|  |  | Linear | 1.18597 | 0.27928 | 4.247 | 0.0000 |
|  |  | Quadratic | -0.07006 | 0.01291 | -5.426 | 0.0000 |
|  |  | Cubic | 0.00141 | 0.00018 | 7.637 | 0.0000 |
|  | High-late Surge | Intercept | 73.89080 | 1.87876 | 39.329 | 0.0000 |
|  |  | Linear | -0.33528 | 0.16908 | -1.983 | 0.0474 |
|  |  | Quadratic | 0.02795 | 0.00356 | 7.851 | 0.0000 |
|  | High-consistent rise | Intercept | 78.73710 | 1.19386 | 65.952 | 0.0000 |
|  |  | Linear | -0.14145 | 0.11239 | -1.259 | 0.2082 |
|  |  | Quadratic | 0.01025 | 0.00247 | 4.159 | 0.0000 |
| MAP | Low-late gradual rise | Intercept | 76.16694 | 2.32708 | 32.731 | 0.0000 |
|  |  | Linear | 1.09970 | 0.35369 | 3.109 | 0.0019 |
|  |  | Quadratic | -0.05909 | 0.01601 | -3.690 | 0.0002 |
|  |  | Cubic | 0.00106 | 0.00022 | 4.731 | 0.0000 |
|  | Low-late rapid rise | Intercept | 75.58220 | 2.04648 | 36.933 | 0.0000 |
|  |  | Linear | 1.67139 | 0.30663 | 5.451 | 0.0000 |
|  | Quadratic | | -0.09145 | 0.01422 -6.431 | | 0.0000 |
|  |  | Cubic | 0.00175 | 0.00020 | 8.556 | 0.0000 |
|  | High-consistent rise | Intercept | 91.98010 | 0.99894 | 92.078 | 0.0000 |
|  |  | Linear | -0.04301 | 0.09379 | -0.459 | 0.6466 |
|  |  | Quadratic | 0.00892 | 0.00202 | 4.423 | 0.0000 |
|  | High-late Surge | Intercept | 96.12299 | 3.83212 | 25.083 | 0.0000 |
|  |  | Linear | -1.77604 | 0.60691 | -2.926 | 0.0034 |
|  |  | Quadratic | 0.10280 | 0.02912 | 3.530 | 0.0004 |
|  |  | Cubic | -0.00107 | 0.00042 | -2.512 | 0.0120 |

**Abbreviations:** BP, blood pressure; SBP, systolic blood pressure; DBP, diastolic blood pressure；MAP, mean arterial

pressure; GH, gestational hypertension.

.

**Table S6**. Model fitting parameters for BP trajectories during pregnancy in women with PE.

| BP | Trajectory name | Parameter | Estimate | Standard Error | T for H_0_:Parameter=0 | Prob > \|T\| |
| --- | --- | --- | --- | --- | --- | --- |
| SBP | Low-stable | Intercept | 113.70927 | 2.22730 | 51.053 | 0.0000 |
|  |  | Linear | -0.56477 | 0.19894 | -2.839 | 0.0045 |
|  |  | Quadratic | 0.01332 | 0.00408 | 3.269 | 0.0011 |
|  | Mid-mid stable | Intercept | 109.28263 | 3.48393 | 31.368 | 0.0000 |
|  |  | Linear | 0.93257 | 0.52301 | 1.783 | 0.0746 |
|  |  | Quadratic | -0.05942 | 0.02401 | -2.475 | 0.0134 |
|  |  | Cubic | 0.00131 | 0.00034 | 3.891 | 0.0001 |
|  | High-consistent rise | Intercept | 116.47964 | 2.56850 | 45.349 | 0.0000 |
|  |  | Linear | 0.15838 | 0.24209 | 0.654 | 0.5130 |
|  |  | Quadratic | 0.01415 | 0.00490 | 2.887 | 0.0039 |
| DBP | Low-stable | Intercept | 64.88373 | 3.07822 | 21.078 | 0.0000 |
|  |  | Linear | 0.93153 | 0.45888 | 2.030 | 0.0424 |
|  |  | Quadratic | -0.06617 | 0.02064 | -3.206 | 0.0014 |
|  |  | Cubic | 0.00123 | 0.00029 | 4.306 | 0.0000 |
|  | Mid-mid stable | Intercept | 69.35995 | 2.40360 | 28.857 | 0.0000 |
|  |  | Linear | 0.63248 | 0.36261 | 1.744 | 0.0812 |
|  |  | Quadratic | -0.04162 | 0.01650 | -2.523 | 0.0117 |
|  |  | Cubic | 0.00097 | 0.00023 | 4.232 | 0.0000 |
|  | High-consistent rise | Intercept | 75.45978 | 2.76005 | 27.340 | 0.0000 |
|  |  | Linear | -0.23118 | 0.26446 | -0.874 | 0.3821 |
|  |  | Quadratic | 0.02283 | 0.00597 | 3.826 | 0.0001 |
| MAP | Low-stable | Intercept | 79.22113 | 3.22567 | 24.560 | 0.0000 |
|  |  | Linear | 0.80777 | 0.48072 | 1.680 | 0.0930 |
|  |  | Quadratic | -0.05607 | 0.02166 | -2.588 | 0.0097 |
|  |  | Cubic | 0.00104 | 0.00030 | 3.458 | 0.0005 |
|  | Mid-mid stable | Intercept | 82.99529 | 2.49749 | 33.232 | 0.0000 |
|  |  | Linear | 0.80204 | 0.38254 | 2.097 | 0.0361 |
|  |  | Quadratic | -0.05145 | 0.01759 | -2.925 | 0.0035 |
|  |  | Cubic | 0.00115 | 0.00025 | 4.668 | 0.0000 |
|  | High-consistent rise | Intercept | 87.19147 | 2.42293 | 35.986 | 0.0000 |
|  |  | Linear | -0.13770 | 0.24231 | -0.568 | 0.5699 |
|  |  | Quadratic | 0.02336 | 0.00528 | 4.422 | 0.0000 |

**Abbreviations:** BP, blood pressure; SBP, systolic blood pressure; DBP, diastolic blood pressure；MAP, mean arterial

pressure; PE, preeclampsia.

**Table S7.** Model fitting parameters for BP trajectories during pregnancy in women with CHTN with superimposed PE.

| BP | Trajectory name | Parameter | Estimate | Standard Error | T for H_0_:Parameter=0 | Prob > \|T\| |
| --- | --- | --- | --- | --- | --- | --- |
| SBP | Low-stable | Intercept | 114.13834 | 3.06753 | 37.209 | 0.0000 |
|  |  | Linear | -0.33827 | 0.27376 | -1.236 | 0.2167 |
|  |  | Quadratic | 0.01334 | 0.00560 | 2.380 | 0.0174 |
|  | Mid-stable | Intercept | 136.30309 | 2.87762 | 47.367 | 0.0000 |
|  |  | Linear | -0.70408 | 0.25698 | -2.740 | 0.0062 |
|  |  | Quadratic | 0.01781 | 0.00529 | 3.368 | 0.0008 |
|  | High-late Surge | Intercept | 153.00394 | 11.72629 | 13.048 | 0.0000 |
|  |  | Linear | 0.48579 | 1.78255 | 0.273 | 0.7852 |
|  |  | Quadratic | -0.10686 | 0.08292 | -1.289 | 0.1976 |
|  |  | Cubic | 0.00270 | 0.00120 | 2.254 | 0.0243 |
| DBP | Low-stable | Intercept | 76.14724 | 2.17147 | 35.067 | 0.0000 |
|  |  | Linear | -0.60888 | 0.19603 | -3.106 | 0.0019 |
|  |  | Quadratic | 0.01649 | 0.00401 | 4.107 | 0.0000 |
|  | Mid-stable | Intercept | 93.47660 | 2.55308 | 36.613 | 0.0000 |
|  |  | Linear | -0.84590 | 0.22465 | -3.765 | 0.0002 |
|  |  | Quadratic | 0.02011 | 0.00455 | 4.420 | 0.0000 |
|  | High-late Surge | Intercept | 77.64964 | 10.44656 | 7.433 | 0.0000 |
|  |  | Linear | 3.77006 | 1.64354 | 2.294 | 0.0219 |
|  |  | Quadratic | -0.22003 | 0.07970 | -2.761 | 0.0058 |
|  |  | Cubic | 0.00392 | 0.00119 | 3.294 | 0.0010 |
| MAP | Low-stable | Intercept | 90.01224 | 2.13877 | 42.086 | 0.0000 |
|  |  | Linear | -0.53721 | 0.19655 | -2.733 | 0.0063 |
|  |  | Quadratic | 0.01554 | 0.00404 | 3.850 | 0.0001 |
|  | Mid-stable | Intercept | 107.99148 | 2.49078 | 43.356 | 0.0000 |
|  |  | Linear | -0.76673 | 0.22057 | -3.476 | 0.0005 |
|  |  | Quadratic | 0.01885 | 0.00451 | 4.177 | 0.0000 |
|  | High-late Surge | Intercept | 107.54343 | 10.37689 | 10.364 | 0.0000 |
|  |  | Linear | 2.12081 | 1.64523 | 1.289 | 0.1975 |
|  |  | Quadratic | -0.16280 | 0.07960 | -2.045 | 0.0410 |
|  |  | Cubic | 0.00332 | 0.00119 | 2.801 | 0.0050 |

**Abbreviations:** BP, blood pressure; SBP, systolic blood pressure; DBP, diastolic blood pressure；MAP, mean arterial pressure; CHTN

with superimposed PE, chronic hypertension with superimposed preeclampsia.

**Abbreviations:** BP, blood pressure; SBP, systolic blood pressure; DBP, diastolic blood pressure；MAP, mean arterial pressure;

CHTN with superimposed PE, chronic hypertension with superimposed. preeclampsia

**Table S8.** Model fitting parameters for BP trajectories during pregnancy in women with CHTN.

| BP | Trajectory name | Parameter | Estimate | Standard Error | T for H_0_:Parameter=0 | Prob > \|T\| |
| --- | --- | --- | --- | --- | --- | --- |
| SBP | Low-stable | Intercept | 124.93729 | 4.70908 | 26.531 | 0.0000 |
|  |  | Linear | 0.80137 | 0.69569 | 1.152 | 0.2494 |
|  |  | Quadratic | -0.05951 | 0.03100 | -1.920 | 0.0549 |
|  |  | Cubic | 0.00106 | 0.00043 | 2.468 | 0.0136 |
|  | Mid-stable | Intercept | 119.39761 | 4.16415 | 28.673 | 0.0000 |
|  |  | Linear | 2.65949 | 0.61769 | 4.306 | 0.0000 |
|  |  | Quadratic | -0.12307 | 0.02787 | -4.416 | 0.0000 |
|  |  | Cubic | 0.00179 | 0.00039 | 4.591 | 0.0000 |
|  | High-consistent rise | Intercept | 142.15424 | 1.90323 | 74.691 | 0.0000 |
|  |  | Linear | 0.46733 | 0.07811 | 5.983 | 0.0000 |
| DBP | Low-stable | Intercept | 80.56722 | 4.19840 | 19.190 | 0.0000 |
|  |  | Linear | 0.59821 | 0.62286 | 0.960 | 0.3369 |
|  |  | Quadratic | -0.05672 | 0.02785 | -2.037 | 0.0417 |
|  |  | Cubic | 0.00106 | 0.00038 | 2.752 | 0.0059 |
|  | Mid-stable | Intercept | 76.44157 | 2.70725 | 28.236 | 0.0000 |
|  |  | Linear | 1.99709 | 0.40537 | 4.927 | 0.0000 |
|  |  | Quadratic | -0.10444 | 0.01839 | -5.680 | 0.0000 |
|  |  | Cubic | 0.00164 | 0.00026 | 6.338 | 0.0000 |
|  | High-consistent rise | Intercept | 91.57060 | 1.37345 | 66.672 | 0.0000 |
|  |  | Linear | 0.31694 | 0.05442 | 5.824 | 0.0000 |
| MAP | Low-stable | Intercept | 96.52643 | 4.09261 | 23.586 | 0.0000 |
|  |  | Linear | 0.69786 | 0.60298 | 1.157 | 0.2472 |
|  |  | Quadratic | -0.06287 | 0.02682 | -2.344 | 0.0191 |
|  |  | Cubic | 0.00116 | 0.00037 | 3.127 | 0.0018 |
|  | Mid-stable | Intercept | 90.09732 | 2.82643 | 31.877 | 0.0000 |
|  |  | Linear | 2.19531 | 0.41820 | 5.249 | 0.0000 |
|  |  | Quadratic | -0.10884 | 0.01889 | -5.762 | 0.0000 |
|  |  | Cubic | 0.00166 | 0.00026 | 6.286 | 0.0000 |
|  | High-consistent rise | Intercept | 108.02071 | 1.40644 | 76.804 | 0.0000 |
|  |  | Linear | 0.35887 | 0.05629 | 6.376 | 0.0000 |

**Abbreviations:** BP, blood pressure; SBP, systolic blood pressure; DBP, diastolic blood pressure；MAP, mean arterial

pressure; CHTN, chronic hypertension

**Abbreviations:** BP, blood pressure; SBP, systolic blood pressure; DBP, diastolic blood pressure；

MAP, mean arterial pressure; CHTN, chronic hypertension.


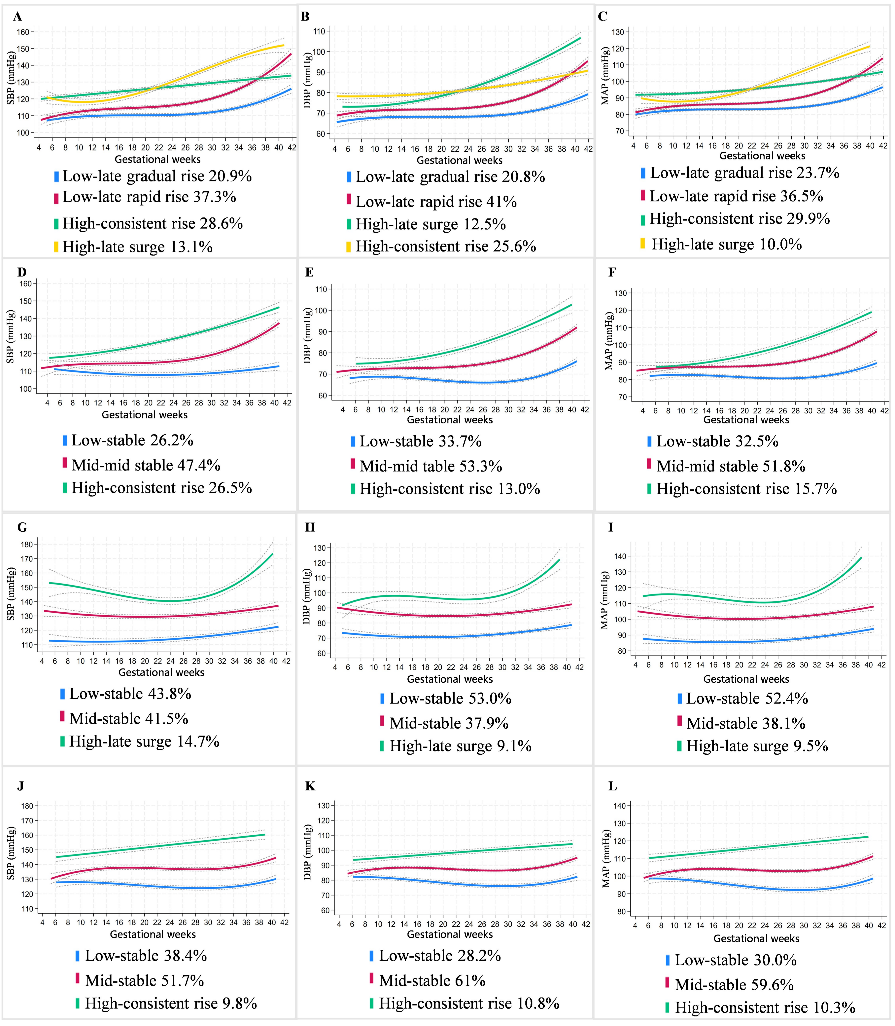


**Figure S2.** Sensitivity analysis of trajectories of SBP, DBP, and MAP during pregnancy in women with HDP.

**Note:**A–C represent the gestational SBP, DBP, and MAP trajectories in women with GH, respectively.
D–F represent the gestational SBP, DBP, and MAP trajectories in women with PE, respectively.
G–I represent the gestational SBP, DBP, and MAP trajectories in women with CHTN with superimposed PE, respectively.
J–L represent the gestational SBP, DBP, and MAP trajectories in women with CHTN, respectively.

**Abbreviations:** SBP, systolic blood pressure; DBP, diastolic blood pressure；MAP, mean arterial pressure; HDP, hypertensive disorders in pregnancy; GH, gestational hypertension; PE, preeclampsia; CHTN with superimposed PE, chronic hypertension with superimposed preeclampsia; CHTN, chronic hypertension.

**Table S9.**  Sensitivity analysis of association between BP trajectories during pregnancy and the risk of unrecovered BP at 6 weeks.

| HDP subtype | BP index | Trajectory group |  | Adjusted model |  |
| --- | --- | --- | --- | --- | --- |
|  |  |  |  | aRR (95%CI) | *P*value |
| GH | SBP | Low-late rapid rise |  | 1.575(0.673-3.683) | 0.295 |
|  |  | High-consistent rise |  | 2.529(1.110-5.765) | 0.027* |
|  |  | High-late surge |  | 4.478(1.863-10.762) | 0.001* |
|  | DBP | Low-late rapid rise |  | 3.068(1.076-8.743) | 0.036* |
|  |  | High-late surge |  | 6.547(2.138-20.043) | 0.001* |
|  |  | High-consistent rise |  | 5.589(1.935-16.142) | 0.001* |
|  | MAP | Low-late rapid rise |  | 2.627(1.010-6.837) | 0.048* |
|  |  | High-consistent rise |  | 4.936(1.931-12.618) | 0.001* |
|  |  | High-late surge |  | 7.018(2.523-19.523) | 0.000* |
| PE | SBP | Mid-mid stable |  | 0.980(0.300-3.202) | 0.973 |
|  |  | High-consistent rise |  | 3.230(1.101-9.477) | 0.033* |
|  | DBP | Mid-mid stable |  | 1.991(0.748-5.294) | 0.168 |
|  |  | High-consistent rise |  | 0.942(0.219-4.051) | 0.936 |
|  | MAP | Mid-mid stable |  | 2.109(0.681-6.531) | 0.196 |
|  |  | High-consistent rise |  | 3.321(0.886-12.453) | 0.075 |
| CHTN with  superimposed PE | SBP | Mid-stable |  | 1.920(0.818-4.504) | 0.134 |
|  |  | High-late surge |  | 2.759(1.068-7.133) | 0.036* |
|  | DBP | Mid-stable |  | 3.386(1.456-7.878) | 0.005* |
|  |  | High-late surge |  | 4.003(1.313-12.207) | 0.015* |
|  | MAP | Mid-stable |  | 2.264(1.022-5.016) | 0.044* |
|  |  | High-late surge |  | 3.976(1.444-10.947) | 0.008* |
| CHTN | SBP | Mid-stable |  | 1.658(0.943-2.915) | 0.079 |
|  |  | High-consistent rise |  | 2.575(1.262-5.250) | 0.009* |
|  | DBP | Mid-stable |  | 2.540(1.226-5.260) | 0.012* |
|  |  | High-consistent rise |  | 3.846(1.668-8.870) | 0.002* |
|  | MAP | Mid-stable |  | 2.056(1.052-4.019) | 0.035* |
|  |  | High-consistent rise |  | 3.648(1.654-8.046) | 0.001* |

**Note:**

**P*≤0.05 was considered statistically significant;

Adjusted for maternal age, education level, gravidity, delivery gestational age, parity status, mode of delivery, neonatal birth weight, and gender

**Abbreviations:** RR risk ratio; 95% CI, 95% confidence interval; BP, blood pressure; HDP, hypertensive disorders in pregnancy; SBP, systolic blood pressure;

DBP, diastolic blood pressure；MAP, mean arterial pressure; GH, gestational hypertension; PE, preeclampsia; CHTN, chronic hypertension; CHTN with

superimposed PE, chronic hypertension with superimposed preeclampsia.

**Table S10**. Statistical description of blood pressure measurement frequency during pregnancy among women with different types of HDP.

| HDP subtype | Gestational stage | Participants | Measurements | Mean | SD | Median |
| --- | --- | --- | --- | --- | --- | --- |
| GH | Overall pregnancy | 1674 | 13154 | 7.86 | 2.79 | 7 |
|  | First trimester | 1674 | 2327 | 1.39 | 0.68 | 1 |
|  | Second trimester | 1674 | 4275 | 2.55 | 1.14 | 2 |
|  | Third trimester | 1674 | 6552 | 3.91 | 1.99 | 3 |
| PE | Overall pregnancy | 607 | 4947 | 8.15 | 2.98 | 7 |
|  | First trimester | 607 | 850 | 1.40 | 0.65 | 1 |
|  | Second trimester | 607 | 1589 | 2.62 | 1.20 | 2 |
|  | Third trimester | 607 | 2508 | 4.13 | 2.14 | 4 |
| CHTN | Overall pregnancy | 635 | 5420 | 8.54 | 3.42 | 8 |
|  | First trimester | 635 | 1025 | 1.61 | 0.97 | 1 |
|  | Second trimester | 635 | 1979 | 3.12 | 1.62 | 3 |
|  | Third trimester | 635 | 2416 | 3.80 | 1.98 | 3 |
| CHTN with superimposed PE | Overall pregnancy | 246 | 2061 | 8.38 | 4.15 | 7 |
|  | First trimester | 246 | 378 | 1.54 | 0.90 | 1 |
|  | Second trimester | 246 | 747 | 3.04 | 2.07 | 3 |
|  | Third trimester | 246 | 936 | 3.80 | 2.33 | 3 |
| Total population | Overall pregnancy | 3162 | 25582 | 8.09 | 3.09 | 7 |
|  | First trimester | 3162 | 4580 | 1.45 | 0.77 | 1 |
|  | Second trimester | 3162 | 8590 | 2.72 | 1.37 | 2 |
|  | Third trimester | 3162 | 12412 | 3.93 | 2.05 | 4 |

**Abbreviations:** GH, gestational hypertension; PE, preeclampsia; CHTN, chronic hypertension; CHTN with superimposed PE, chronic hypertension with superimposed

preeclampsia; SD, Standard Deviation.

**Table S11.** Summary of BP measurement frequency by HDP subtype (to be included in Supplementary Material **Table S10.**)

| HDP subtype | Average measurements per woman  (Overall pregnancy) | Measurements in 1st trimester (Mean) | Measurements in 2nd trimester (Mean) | Measurements in 3srd trimester (Mean) |
| --- | --- | --- | --- | --- |
| GH | 7.86 | 1.39 | 2.55 | 3.91 |
| PE | 8.15 | 1.40 | 2.62 | 4.13 |
| CHTN | 8.54 | 1.61 | 3.12 | 3.80 |
| CHTN with superimposed PE | 8.38 | 1.54 | 3.04 | 3.80 |

**Abbreviations:** GH, gestational hypertension; PE, preeclampsia; CHTN, chronic hypertension; CHTN with superimposed PE, chronic hypertension

with superimposed preeclampsia.
